# Telocytes are reduced during fibrotic remodelling of the colonic wall in ulcerative colitis

**DOI:** 10.1111/jcmm.12457

**Published:** 2014-10-06

**Authors:** Mirko Manetti, Irene Rosa, Luca Messerini, Lidia Ibba-Manneschi

**Affiliations:** a Department of Experimental and Clinical Medicine, Section of Anatomy and Histology, University of FlorenceFlorence, Italy; b Department of Experimental and Clinical Medicine, Section of Surgery, Histopathology and Molecular Pathology, University of FlorenceFlorence, Italy

**Keywords:** telocytes, ulcerative colitis, colonic wall, interstitial cells of Cajal, myofibroblasts, fibrosis, CD34, immunohistochemistry

## Abstract

Ulcerative colitis (UC) is characterized by chronic relapsing intestinal inflammation finally leading to extensive tissue fibrosis and resulting in a stiff colon unable to carry out peristalsis or to resorb fluids. Telocytes, a peculiar type of stromal cells, have been recently identified in the human gastrointestinal tract. Several roles have been proposed for telocytes, including mechanical support, intercellular signalling and modulation of intestinal motility. The aim of the present work was to investigate the presence and distribution of telocytes in colonic specimens from UC patients compared with controls. Archival paraffin-embedded samples of the left colon from UC patients who underwent elective bowel resection and controls were collected. Tissue sections were stained with Masson's trichrome to detect fibrosis. Telocytes were identified by CD34 immunohistochemistry. In early fibrotic UC cases, fibrosis affected the muscularis mucosae and submucosa, while the muscularis propria was spared. In advanced fibrotic UC cases, fibrosis extended to affect the muscle layers and the myenteric plexus. Few telocytes were found in the muscularis mucosae and submucosa of both early and advanced fibrotic UC colonic wall. In the muscle layers and myenteric plexus of early fibrotic UC, telocytes were preserved in their distribution. In the muscularis propria of advanced fibrotic UC, the network of telocytes was reduced or even completely absent around smooth muscle bundles and myenteric plexus ganglia, paralleling the loss of the network of interstitial cells of Cajal. In UC, a loss of telocytes accompanies the fibrotic remodelling of the colonic wall and might contribute to colonic dysmotility.

## Introduction

Inflammatory bowel diseases, including Crohn's disease (CD) and ulcerative colitis (UC), are complex diseases in which the interaction of genetic, environmental and microbial factors drives chronic relapsing and remitting intestinal inflammation that finally leads to extensive tissue fibrosis [1,2]. This is particularly relevant for small bowel CD. Indeed, in UC the deposition of the extracellular matrix is mainly restricted to the mucosal and submucosal layers of the large bowel, while in CD fibrosis commonly involves the entire bowel wall, including the mucosa, submucosa, muscularis propria and subserosa layers, and can result in critical narrowing of the lumen and strictures or stenosis, leading to intestinal obstruction that requires surgery [3]. Accordingly, the frequency of benign stenosis in UC is much lower than in CD, reported as being 3.2–11.2%, with fibrosis in the submucosa or deeper pointed out as one of the causes [4]. However, increasing evidence indicates that the development of intestinal fibrosis in UC is a neglected problem which has remained largely unexplored [5]. In UC, fibrosis results in a stiff, fibrotic colon unable to carry out peristalsis or to resorb fluids – the major tasks of the large bowel, contributing to the abdominal pain and diarrhoea commonly experienced by patients with active disease or even in remission [6–8]. Colonic dysmotility in UC patients has been well-established and has been attributed to alterations of the enteric neuromuscular compartment, such as a reduced density of myenteric neurons, glial cells and interstitial cells of Cajal (ICC) [9,10].

Telocytes are a peculiar type of stromal (interstitial) cells recently identified in a variety of human tissues and organs, including the gastrointestinal tract [11–15]. Telocytes are characterized by a small cell body and extremely long, thin and varicose cytoplasmic processes, termed telopodes, forming a three-dimensional network that may function as a scaffold to define the correct organization of tissues and organs during pre-natal life or their repair/renewal in post-natal life [11–13,16]. According to their specific locations within different organs, several roles have been proposed for telocytes, including mechanical support, intercellular signalling, either by cell-to-cell contacts or by secreting paracrine signalling molecules, immune surveillance, tissue regeneration by cooperating with resident mesenchymal stem cell niches, and modulation of intestinal motility by spreading the slow waves generated by the pacemaker ICC [11–13,15–27].

In a recent study, we have demonstrated that telocytes are reduced during fibrotic remodelling of the intestinal wall in CD patients, suggesting that the loss of telocytes might contribute to bowel architectural derangement and dysmotility [28]. On the basis of these observations, we designed the present work with the purpose of investigating the presence and distribution of telocytes in colonic specimens from UC patients.

## Materials and methods

### Tissue samples, histochemistry and histopathological analysis

The study was entirely carried out on paraffin-embedded tissue samples collected at the archives of the Section of Surgery, Histopathology and Molecular Pathology and the Section of Anatomy and Histology, Department of Experimental and Clinical Medicine, University of Florence. Full-thickness archival samples of left (descending and sigmoid) colon were obtained from 12 patients with UC (6 males, 6 females; age range 46–64 years) who had undergone elective bowel resection because of left-sided colitis lasting over 5 years. All UC patients had been scheduled for surgical intervention owing to a persistent condition of refractoriness to immunosuppressant therapy and/or steroid dependence. Four full-thickness paraffin blocks from each UC patient were collected. Archival colonic samples from 10 patients (5 males, 5 females; age range 44–65 years), who had undergone surgery for uncomplicated left colon cancer and without previous history of abdominal surgery, inflammatory bowel disease or intestinal obstruction, served as controls. Specimens were taken at least 8 cm away from any macroscopically visible lesion. Three full-thickness paraffin blocks were obtained from each control patient. All the patients had signed a written informed consent form and the Institutional Review Board approved the study.

Tissue sections were cut (5 μm thick) using a Leica RM2255 rotary microtome (Leica Microsystems, Mannheim, Germany), deparaffinized, rehydrated and stained with haematoxylin and eosin for routine histology and with Masson's trichrome with aniline blue (catalogue no. 04-010802; Bio-Optica, Milan, Italy) to detect fibrosis. For both histochemical stains, three sections from each paraffin block were examined per patient. Tissue sections were observed under a Leica DM4000 B microscope (Leica Microsystems), and transmitted light images were captured using a Leica DFC310 FX 1.4-megapixel digital colour camera equipped with the Leica software application suite LAS V3.8 (Leica Microsystems). The morphological features of the colon were carefully assessed on haematoxylin and eosin-stained specimens by an experienced pathologist (L.M.). The microscopic analysis of samples obtained from all UC patients confirmed the presence of classical mucosal/submucosal histopathological lesions consistent with UC. A normal morphology was detected in colonic samples from all control patients.

### Immunoperoxidase-based immunohistochemistry

Immunohistochemistry was performed with an indirect immunoperoxidase-based method. After deparaffinization, tissue sections (5 μm thick) were boiled for 10 min. in sodium citrate buffer (10 mM, pH 6.0) for antigen retrieval and treated with 3% H_2_O_2_ in methanol for 15 min. at room temperature to block endogenous peroxidase activity. Sections were then washed and incubated with Ultra V block (UltraVision Large Volume Detection System Anti-Polyvalent, HRP, catalogue no. TP-125-HL; LabVision, Fremont, CA, USA) for 10 min. at room temperature according to the manufacturer's protocol. After blocking non-specific site binding, slides were incubated overnight at 4°C with mouse monoclonal anti-human CD34 antibody (1:50 dilution; clone QBEnd-10, catalogue no. M7165; Dako, Glostrup, Denmark) diluted in 1% bovine serum albumin (BSA) in PBS. The day after, tissue sections were washed in PBS and incubated with biotinylated secondary antibodies (UltraVision Large Volume Detection System Anti-Polyvalent, HRP; LabVision) for 10 min. at room temperature. Subsequently, the slides were washed three times in PBS and incubated with streptavidin peroxidase (UltraVision Large Volume Detection System Anti-Polyvalent, HRP; LabVision) for 10 min. at room temperature. Immunoreactivity was developed using 3-amino-9-ethylcarbazole (AEC kit, catalogue no. TA-125-SA; LabVision) as chromogen (brownish-red colour). Sections were finally counterstained with haematoxylin, washed, mounted in an aqueous mounting medium and observed under a Leica DM4000 B microscope equipped with fully automated transmitted light axes (Leica Microsystems). Sections not exposed to primary antibodies or incubated with isotype-matched and concentration-matched non-immune mouse IgG (Sigma-Aldrich, St. Louis, MO, USA) were included as negative controls for antibody specificity.

### Immunofluorescence staining

Paraffin-embedded tissue sections (5 μm thick) were deparaffinized and boiled for 10 min. in sodium citrate buffer (10 mM, pH 6.0). Sections were washed in PBS, incubated in 2 mg/ml glycine for 10 min. to quench autofluorescence caused by free aldehydes, and then blocked for 1 hr at room temperature with 1% BSA in PBS. For double immunofluorescence staining, the sections were incubated overnight at 4°C with a mixture of mouse monoclonal anti-human CD34 antibody (1:50 dilution; clone QBEnd-10, catalogue no. M7165; Dako) and rabbit polyclonal anti-human CD31/platelet-endothelial cell adhesion molecule-1 (PECAM-1; 1:50 dilution; catalogue no. ab28364; Abcam, Cambridge, UK), rabbit polyclonal anti-c-kit/CD117 (1:200 dilution; catalogue no. A4502; Dako) or rabbit polyclonal anti-α-smooth muscle actin (α-SMA; 1:100 dilution; catalogue no. ab5694; Abcam) antibody diluted in PBS with 1% BSA. The day after, the slides were washed three times in PBS and incubated for 45 min. at room temperature in the dark with a mixture of Alexa Fluor-488-conjugated goat antimouse IgG and Rhodamine Red-X-conjugated goat anti-rabbit IgG (Invitrogen, San Diego, CA, USA) diluted 1:200 in PBS with 1% BSA. Irrelevant isotype-matched and concentration-matched mouse and rabbit IgG (Sigma-Aldrich) were used as negative controls. Cross-reactivity of secondary antibodies was tested in control experiments in which primary antibodies were omitted. Nuclei were counterstained with 4′,6-diamidino-2-phenylindole (DAPI; Chemicon International, Temecula, CA, USA). Tissue sections were then mounted with an antifade aqueous mounting medium (Biomeda Gel Mount; Electron Microscopy Sciences, Foster City, CA, USA) and examined with a Leica DM4000 B microscope equipped with fully automated fluorescence axes (Leica Microsystems). Fluorescence images were captured with a Leica DFC310 FX 1.4-megapixel digital colour camera equipped with the Leica software application suite LAS V3.8 (Leica Microsystems).

### Quantitative analysis

Quantitative analysis of telocytes was performed on colonic sections double-immunostained with the mouse monoclonal anti-CD34 antibody and the rabbit polyclonal anti-CD31/PECAM-1 antibody and counterstained with DAPI for nuclei. CD34-positive/CD31-negative telocytes were counted on digitized images captured from at least six randomly chosen microscopic fields (40× original magnification) of the muscularis mucosae, as well as of the submucosa, circular muscle layer, myenteric plexus and longitudinal muscle layer per section. For every study patient, two sections from each paraffin block were examined. Only the cells with well-defined nuclei were counted. The same procedure was used for the quantification of submucosal myofibroblasts (α-SMA-positive spindle-shaped cells) on sections immunolabelled with the rabbit polyclonal anti-α-SMA antibody and counterstained with DAPI for nuclei, as well as for the quantification of ICC (c-kit-positive cells with typical ICC morphology) in circular muscle layer, myenteric plexus and longitudinal muscle layer on sections immunolabelled with the rabbit polyclonal anti-c-kit/CD117 antibody and counterstained with DAPI. Counting was performed by two independent observers (M.M. and L.I.-M.) who were blinded with regard to the sample classification. The final results were expressed as percent change in cell counts, where mean values in control samples were arbitrarily assumed as 100%.

### Statistical analysis

All data are represented as the mean and SEM. Statistical analysis was performed by using the Student's *t*-test for independent samples. *P* < 0.05 was considered statistically significant. The SPSS software for Windows Version 12.0 (SPSS, Chicago, IL, USA) was used.

## Results

**Fig. 1:**
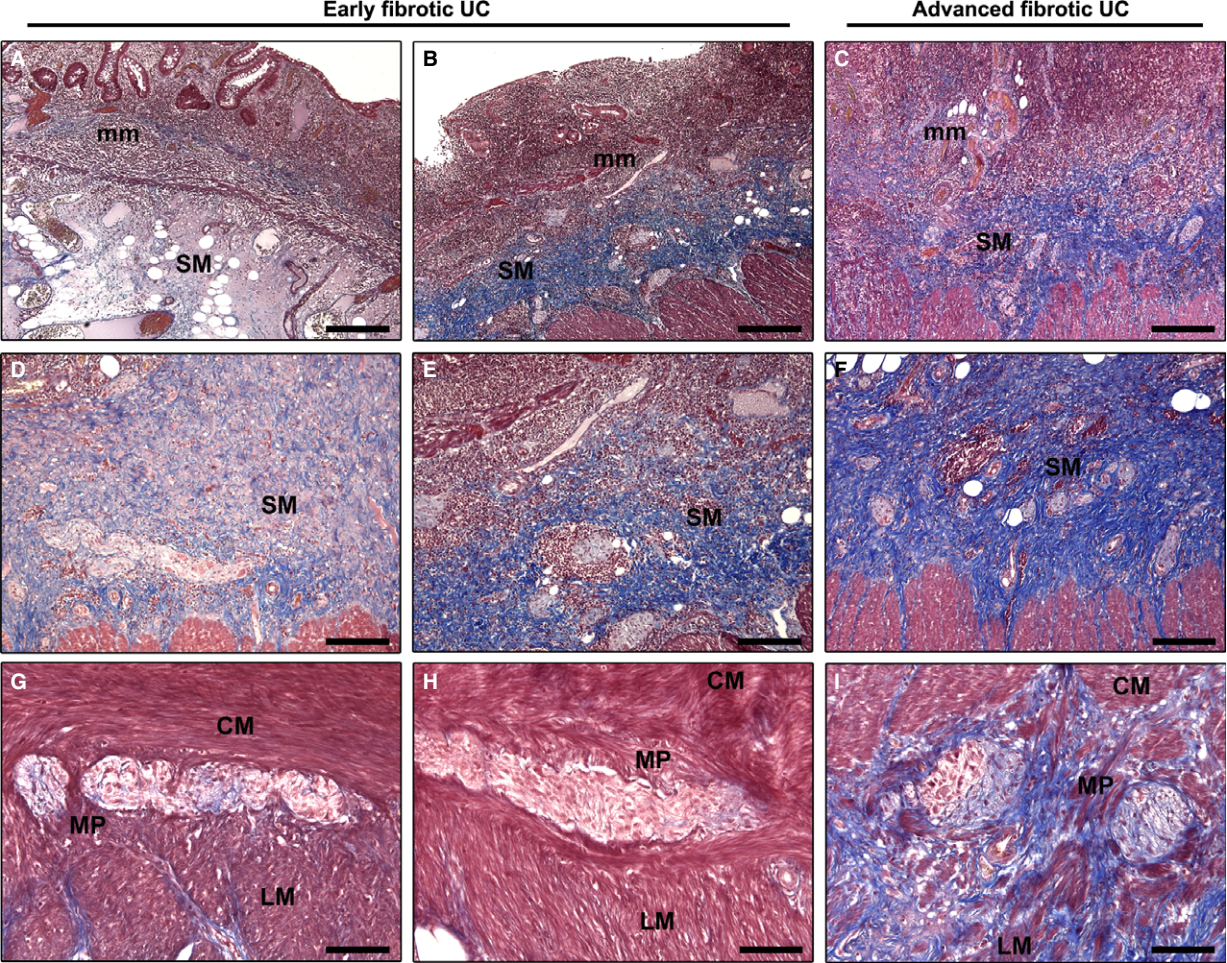
Masson's trichrome-stained sections of colonic wall from patients with ulcerative colitis (UC). Representative microphotographs of sections from UC patients in an early-phase (**A**, **B**, **D**, **E**, **G** and **H**) or an advanced phase (**C**, **F** and **I**) of fibrotic remodelling of the colonic wall are shown. (**A**–**C**) Low magnification views of the colonic mucosa and submucosa show the presence of histopathological lesions typical of UC, such as surface epithelial damage, erosions, goblet cell depletion, cryptitis, crypt abscesses, distortion and/or destruction and a marked infiltration of inflammatory and immune cells throughout the muscularis mucosae and submucosa. (**D**–**F**) Submucosa. (**G**–**I**) Muscle layers and myenteric plexus. In early fibrotic UC cases, fibrosis affects the muscularis mucosae (**A** and **B**), while the submucosa is characterized by the presence of areas displaying oedema and a pattern of incoming fibrosis (**A** and **D**) and areas of established fibrosis (**B** and **E**). Note collagen fibres surrounded by oedema in an area of the submucosa (**D**), while tightly packed collagen bundles are visible in another area (**E**). No fibrosis is observed in the muscularis propria (**G** and **H**). In advanced fibrotic UC cases, an increased deposition of extracellular matrix is observed in the muscularis mucosae, which appears markedly thickened (**C**), widespreadly in the submucosal layer (**C** and **F**), and also in wide areas of the muscle layers and myenteric plexus (**I**). Note the accumulation of dense collagen bundles surrounding myenteric ganglia (**I**). All tissue sections are stained with Masson's trichrome with aniline blue (red colour: cytoplasm; blue colour: collagen). mm: muscularis mucosae; SM: submucosa; CM: circular muscle layer; MP: myenteric plexus; LM: longitudinal muscle layer. Scale bar = 400 μm (**A**–**C**), 200 μm (**D**–**F**), 100 μm (**G**–**I**).

The presence and distribution of telocytes were investigated in full-thickness biopsies of the left colon obtained from UC patients and controls. The histopathological analysis of surgical samples obtained from all UC patients confirmed the presence of mucosal/submucosal lesions typical of UC, including erosions, widespread surface epithelial damage, goblet cell depletion, cryptitis, frank crypt abscesses, crypt distortion and/or destruction, as well as a marked infiltration of inflammatory and immune cells in the muscularis mucosae and submucosa (Fig. 1A–C). Based on the microscopic examination of Masson's trichrome-stained tissue sections, UC specimens were categorized in an early-phase (*n* = 7) or an advanced phase (*n* = 5) of fibrotic remodelling of the colonic wall. In early fibrotic UC cases, fibrosis affected the muscularis mucosae and submucosa, while the muscularis propria was spared (Fig. 1A, B, D, E, G and H). In particular, the submucosa was characterized by the presence of areas displaying oedema and a pattern of incoming fibrosis (Fig. 1A and D) which were abruptly mixed with areas displaying established fibrosis with abundant and closely packed collagen bundles (Fig. 1B and E). In advanced fibrotic UC cases, an increased deposition of the extracellular matrix was observed in the muscularis mucosae, which appeared markedly thickened, and widespread in the submucosa (Fig. 1C and F). Moreover, in advanced fibrotic UC cases, fibrosis extended to involve also wide areas of the circular and longitudinal muscle layers and the myenteric plexus (Fig. 1I).

**Fig. 2:**
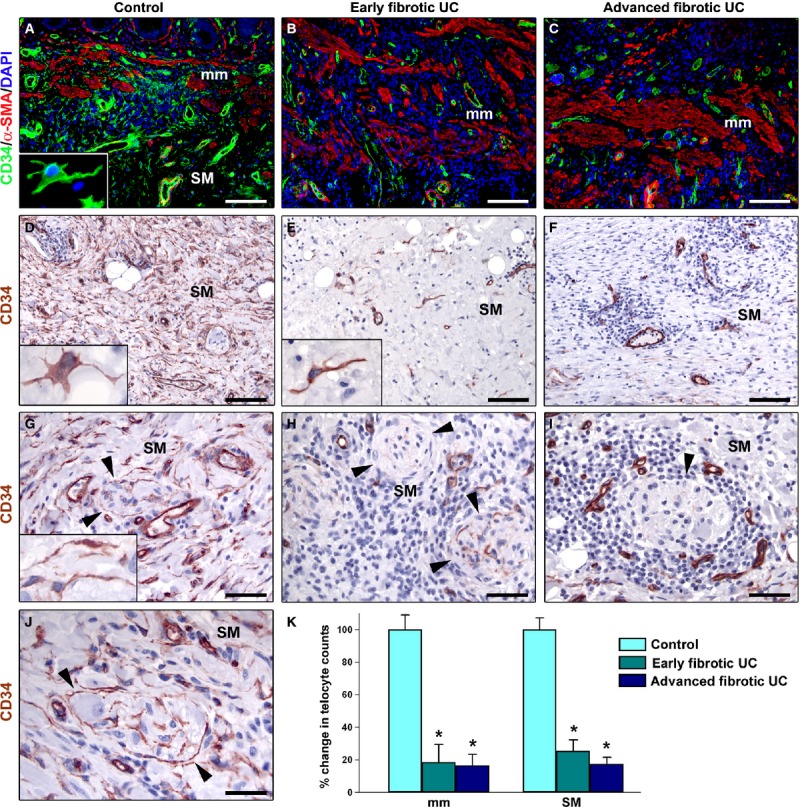
Muscularis mucosae and submucosa of colonic wall specimens from controls (**A**, **D**, **G** and **J**), early fibrotic ulcerative colitis (UC) cases (**B**, **E** and **H**) and advanced fibrotic UC cases (**C**, **F** and **I**). (**A–C**) Double immunofluorescence labelling for CD34 (green) and α-smooth muscle actin (α-SMA, red) with DAPI (blue) counterstain for nuclei. (**D–J**) CD34 immunoperoxidase labelling (brownish-red) with haematoxylin counterstain. (**A**) In the muscularis mucosae of control colonic wall, numerous telocytes are present among smooth muscle bundles. Smooth muscle cells are α-SMA-positive. *Inset*: Higher magnification view of a telocyte located in the muscularis mucosae displaying a slender nucleated body and long, thin and varicose processes. (**B** and **C**) In the muscularis mucosae of early fibrotic UC (**B**) and advanced fibrotic UC (**C**) colonic wall, very few or no telocytes are observed among smooth muscle bundles. CD34 immunopositivity can be observed only in vessels. Note that the muscularis mucosae appears markedly thickened and infiltrated by inflammatory and immune cells. (**D**, **G** and **J**) In the submucosa of control samples, telocytes form an abundant network and surround vessels and ganglia (*arrowheads* in **G** and **J**). *Insets* in **D** and **G**: Representative higher magnification views of submucosal telocytes with long and varicose telopodes. (**E**, **F**, **H** and **I**) In the submucosa of early fibrotic UC (**E** and **H**) and advanced fibrotic UC (**F** and **I**) specimens, telocytes are severely reduced or even completely undetectable. Note a telocyte dispersed in the submucosal matrix and surrounded by some inflammatory cells (*inset* in **E**). Few or no telocytes can be observed around submucosal ganglia surrounded by the inflammatory infiltrate (*arrowheads* in **H** and **I**). Scale bar = 100 μm (**A–F**), 50 μm (**G–I**), 30 μm (**J**). (**K**) Results of quantitative analysis of telocytes in the muscularis mucosae and submucosa of controls (*n* = 10), early fibrotic UC (*n* = 7) and advanced fibrotic UC (*n* = 5) patients. Data are mean values and SEM. *P < 0.05 *versus* control. mm: muscularis mucosae; SM: submucosa.

**Fig. 3:**
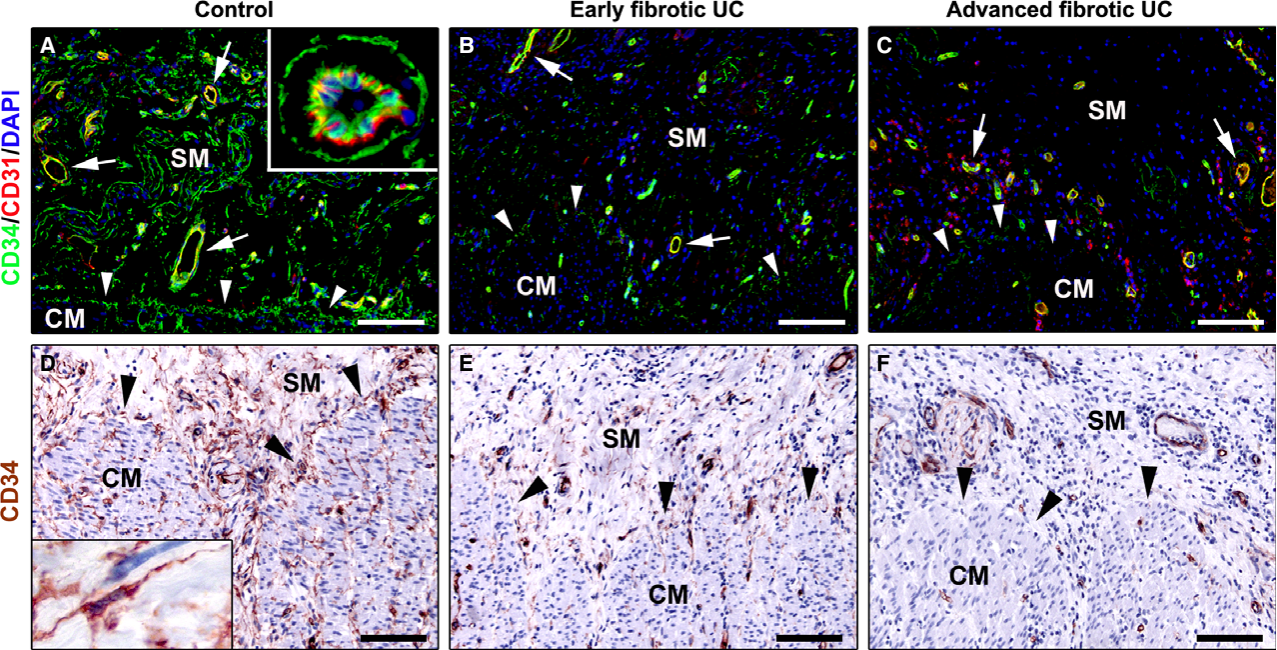
Submucosa and submucosal border of the circular muscle layer of colonic wall specimens from controls (**A** and **D**), early fibrotic ulcerative colitis (UC) cases (**B** and **E**) and advanced fibrotic UC cases (**C** and **F**). (**A–C**) Double immunofluorescence labelling for CD34 (green) and the pan-endothelial cell marker CD31 (red) with DAPI (blue) counterstain for nuclei. Telocytes are CD34-positive and CD31-negative, while vascular endothelial cells are CD34/CD31-double-positive (*arrows*). (**D–F**) CD34 immunoperoxidase labelling (brownish-red) with haematoxylin counterstain. Numerous telocytes are present throughout the submucosa of control colonic wall (**A** and **D**), while they are very few or absent in the submucosa of both early fibrotic (**B** and **E**) and advanced fibrotic (**C** and **F**) UC cases. *Inset* in **A**: Higher magnification view of a submucosal blood vessel encircled by telocyte prolongations. In control specimens, telocytes form an almost continuous layer at the submucosal border of the muscularis propria (*arrowheads* in **A** and **D**). *Inset* in **D**: Higher magnification view of a submucosal telocyte bordering the circular muscle layer with two long and varicose processes. The layer of telocytes at the submucosal border of the muscularis propria is discontinuous in early fibrotic UC cases (*arrowheads* in **B** and **E**) or completely undetectable in advanced fibrotic UC cases (*arrowheads* in **C** and **F**). SM: submucosa; CM: circular muscle layer. Scale bar = 100 μm (**A–F**).

According to previously published studies [14,15,28–30], telocytes were identified by light and fluorescence microscopy using CD34 immunostaining. The distribution of telocytes in control specimens was consistent with that reported in the literature [14,15,28]. In control colonic wall, telocytes were numerous in the muscularis mucosae, where they were distributed among smooth muscle bundles (Fig. 2A), and in the submucosa, where they formed a three-dimensional network surrounding vessels and ganglia (Figs 2A, D, G and J, 3A), as well as an almost continuous layer at the submucosal border of the muscularis propria (Fig. 3A and D). These cells showed a slender nucleated body and a variable number of long, thin and varicose processes, the telopodes (Figs 2A, D and G insets, J and 3A and D insets). In agreement with previous reports [15,28,29], telocytes were CD34-positive and CD31-negative, and therefore they could be clearly distinguished from CD34/CD31-double-positive vascular endothelial cells (Fig. 3A and inset).

Both in early and advanced fibrotic UC colonic wall, very few or no telocytes were observed among smooth muscle bundles of the muscularis mucosae, which appeared thickened and infiltrated by inflammatory and immune cells (Fig. 2B and C). Moreover, telocytes were severely reduced or even undetectable in both the oedematous and fibrotic areas of submucosa of early fibrotic UC cases, as well as in the submucosa of advanced fibrotic UC cases (Figs 2E, F, H and I, 3B and C). As shown in Figure 2H and I, in both early and advanced fibrotic UC submucosa telocytes were very few or absent around ganglia. At the submucosal border of the muscularis propria, the layer of telocytes was discontinuous in early fibrotic UC cases or even completely absent in advanced fibrotic UC cases (Fig. 3B, C, E and F). Quantitative analysis revealed that telocytes were significantly reduced in the muscularis mucosae and submucosa of both early and advanced fibrotic UC cases compared with controls (Fig. 2K).

**Fig. 4:**
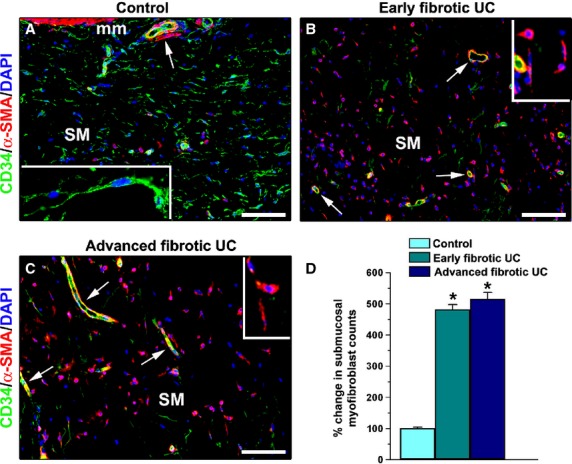
Submucosa of colonic wall specimens from controls (**A**), early fibrotic ulcerative colitis (UC) cases (**B**) and advanced fibrotic UC cases (**C**). (**A**–**C**) Double immunofluorescence labelling for CD34 (green) and α-smooth muscle actin (α-SMA, red) with DAPI (blue) counterstain for nuclei. (**A**) In the submucosa of control specimens, a network of CD34-positive telocytes is visible, while very few α-SMA-positive spindle-shaped cells (myofibroblasts) are present. *Inset* in **A**: Higher magnification view of a telocyte with two long varicose processes. In the submucosa of both early fibrotic (**B**) and advanced fibrotic (**C**) UC cases, there are few or no telocytes, whereas α-SMA-positive myofibroblasts are numerous. As expected, in all colonic sections α-SMA immunopositivity is also observed in vascular wall pericytes and smooth muscle cells (*arrows*). *Insets* in **B** and **C**: Representative higher magnification views of submucosal myofibroblasts. mm: muscularis mucosae; SM: submucosa. Scale bar = 100 μm (**A**–**C**). (**D**) Results of quantitative analysis of submucosal myofibroblasts in controls (*n* = 10), early fibrotic UC (*n* = 7) and advanced fibrotic UC (*n* = 5) patients. Data are mean values and SEM. *P < 0.05 *versus* control.

In the submucosa of all UC specimens, CD34/α-SMA double immunolabelling not only demonstrated the loss of telocytes but it also revealed the presence of numerous α-SMA-positive spindle-shaped myofibroblasts (Fig. 4B, C and insets), which are considered the cell type mostly responsible for the excessive synthesis and deposition of the extracellular matrix during fibrosis [31]. On the contrary, in the submucosa of control colonic specimens many CD34-positive telocytes were present, while myofibroblasts were very few or absent (Fig. 4A and inset). As shown in Figure 4D, the number of myofibroblasts was significantly increased in the submucosa of both early and advanced fibrotic UC cases compared with controls, without any significant difference between the two UC groups.

**Fig. 5:**
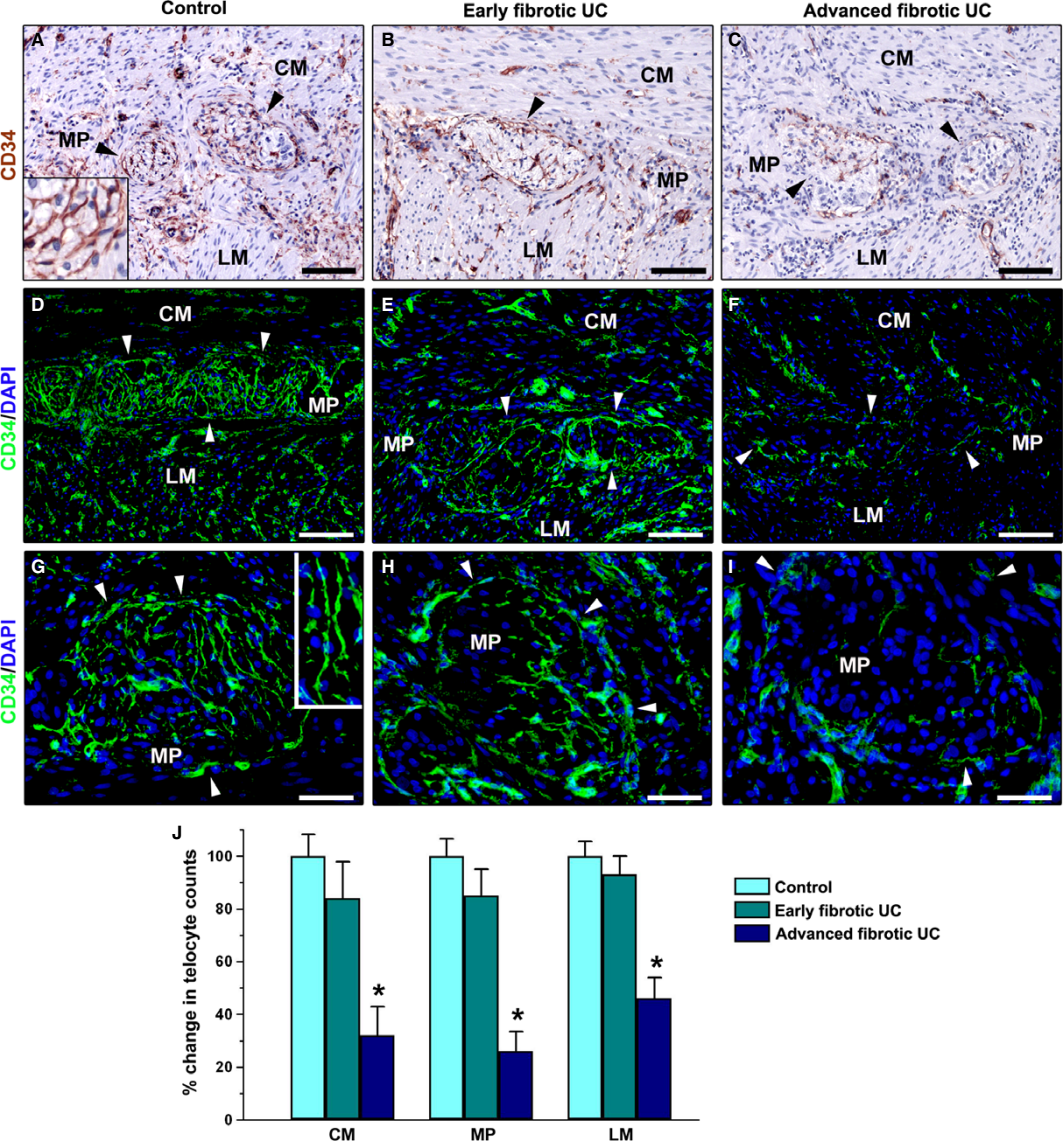
Muscularis propria of colonic wall specimens from controls (**A**, **D** and **G**), early fibrotic ulcerative colitis (UC) cases (**B**, **E** and **H**) and advanced fibrotic UC cases (**C**, **F** and **I**). (**A–C**) CD34 immunoperoxidase labelling (brownish-red) with haematoxylin counterstain. (**D–I**) Immunofluorescence labelling for CD34 (green) with DAPI (blue) counterstain for nuclei. (**A**, **D** and **G**) In control colonic sections, telocytes are numerous among smooth muscle bundles of the circular and longitudinal muscle layers, form a network around the myenteric ganglia (*arrowheads*) and are also present in the interganglionic region of the myenteric plexus. *Insets* in **A** and **G**: Higher magnification views of telocytes enveloping ganglia with their long and varicose processes. (**B**, **E** and **H**) In both muscle layers and myenteric plexus region of early fibrotic UC cases, telocyte distribution is similar to that observed in control samples. Note the abundant network of telocytes around myenteric ganglia (*arrowheads*). (**C**, **F** and **I**) In advanced fibrotic UC cases, very few telocytes are visible in fibrotic areas of muscle layers, and the telocyte network is discontinuous or even almost absent around ganglia (*arrowheads*) and in the interganglionic region of the myenteric plexus. Scale bar = 100 μm (**A–F**), 50 μm (**G–I**). (**J**) Results of quantitative analysis of telocytes in the circular muscle layer, myenteric plexus region and longitudinal muscle layer of controls (*n* = 10), early fibrotic UC (*n* = 7) and advanced fibrotic UC (*n* = 5) patients. Data are mean values and SEM. *P < 0.05 *versus* control. CM: circular muscle layer; MP: myenteric plexus; LM: longitudinal muscle layer.

In the muscularis propria of control colonic sections, telocytes were broadly distributed among smooth muscle cells and bundles within the circular and longitudinal muscle layers, and in the myenteric plexus region (Fig. 5A, D and G). In particular, telocytes formed an abundant network enveloping myenteric ganglia and were also present along the nerve bundles of the interganglionic region (Fig. 5A, D, G and insets). In the muscle layers and myenteric plexus of early fibrotic UC cases, telocytes were mainly preserved in their distribution (Fig. 5B, E and H). Conversely, in advanced fibrotic UC cases, the intramuscular telocytes were severely reduced in the fibrotic areas of muscle layers, and the network of telocytes was discontinuous or even almost completely absent around myenteric plexus ganglia and in the interganglionic region (Fig. 5C, F and I). Quantitative analysis confirmed that in both circular and longitudinal muscle layers and in the myenteric plexus of advanced fibrotic UC cases telocytes were significantly decreased compared with control colonic specimens, while no significant difference in intramuscular telocytes was found between early fibrotic UC and the control group (Fig. 5J).

**Fig. 6:**
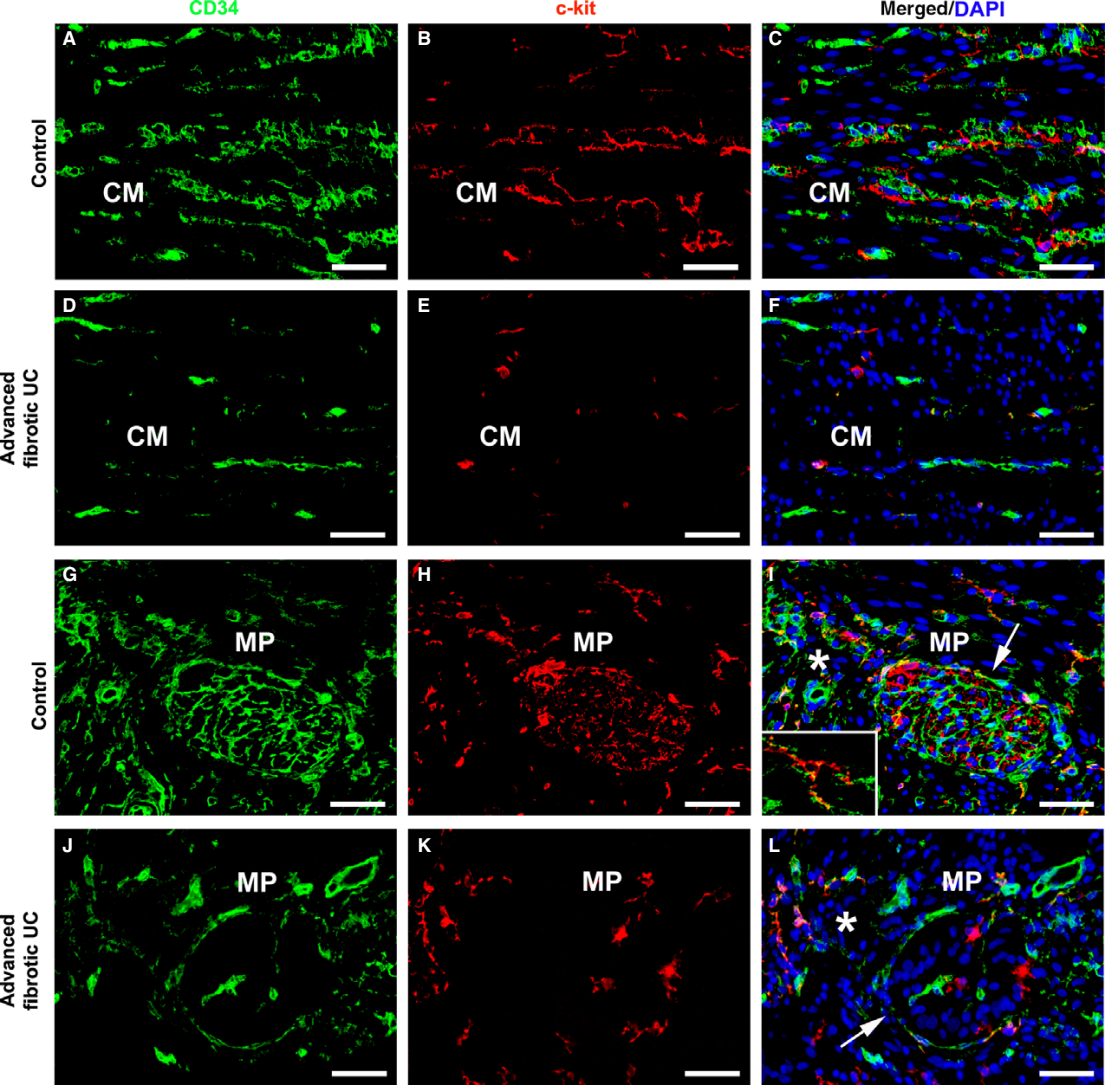
Muscularis propria of colonic wall specimens from controls **(A–C** and **G–I**) and advanced fibrotic ulcerative colitis (UC) cases (**D–F** and **J–L**). (**A–L**) Double immunofluorescence labelling for CD34 (green) and c-kit/CD117 (red) with DAPI (blue) counterstain for nuclei. Telocytes are CD34-positive/c-kit-negative, whereas interstitial cells of Cajal (ICC) are c-kit-positive/CD34-negative. (**A–C**) In muscle layers of control colonic sections, telocytes and ICC form interconnected networks among smooth muscle bundles. (**D–F**) In muscle layers of advanced fibrotic UC cases, very few telocytes and ICC can be observed. Representative microphotographs of the circular muscle layer are shown. (**G–I**) Note the abundant networks of telocytes and ICC around ganglia (*arrow* in **I**) and in the interganglionic region (*asterisk* in **I**) of the myenteric plexus of control colonic wall. *Inset* in **I**: Higher magnification view of an ICC surrounded by telocyte prolongations. (**J–L**) In advanced fibrotic UC cases, both telocytes and ICC are scarce around myenteric ganglia (*arrow* in **L**) and in the interganglionic region (*asterisk* in **L**). CM: circular muscle layer; MP: myenteric plexus. Scale bar = 50 μm (**A–L**).

**Fig. 7:**
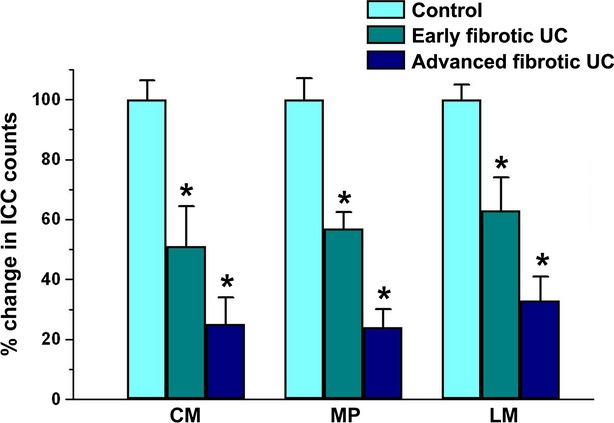
Results of quantitative analysis of interstitial cells of Cajal (ICC) in the circular muscle layer, myenteric plexus region and longitudinal muscle layer of controls (*n* = 10), early fibrotic UC (*n* = 7) and advanced fibrotic UC (*n* = 5) patients. Data are mean values and SEM. *P < 0.05 *versus* control. CM: circular muscle layer; MP: myenteric plexus; LM: longitudinal muscle layer.

To simultaneously identify telocytes and ICC in the muscularis propria of the colonic wall, we performed double immunofluorescence staining for CD34 and c-kit/CD117. According to previous reports [14,15,28], ICC were c-kit-positive and CD34-negative (Fig. 6). CD34/c-kit double immunolabelling further revealed that the loss of telocytes was paralleled by the loss of the ICC network both in muscle layers and myenteric plexus region of advanced fibrotic UC cases compared with controls (Fig. 6). Moreover, quantitative analysis showed that, at variance with telocytes, intramuscular and myenteric ICC were significantly reduced not only in advanced fibrotic UC cases but also in the early fibrotic UC group, compared with controls (Fig. 7).

## Discussion

In this study, we report the novel finding that telocytes are involved in the pathological processes of UC. Indeed, our data clearly show that a progressive reduction and loss of telocytes accompany the fibrotic remodelling of the colonic wall in UC patients, mirroring the pathological findings recently described in the terminal ileum of patients affected by small bowel CD [28].

Intestinal fibrosis is considered a common complication of inflammatory bowel disease and can occur both in UC and CD, closely following the distribution and location of inflammation [1–5,32]. In fact, there is evidence that chronic exposure of intestinal fibroblasts to inflammatory mediators may drive their transition to activated α-SMA-expressing myofibroblasts with consequent abnormal collagen production and tissue remodelling necessary for the initiation of the fibrotic process [32,33]. However, it also appears that inflammation subsequently plays a minor role in fibrosis progression and, accordingly, anti-inflammatory treatment in inflammatory bowel diseases may not be able to limit fibrosis once excessive extracellular matrix deposition has started [3]. Hence, fibrotic remodelling may lead to profound and often irreversible changes, potentially affecting any cell type of the stromal compartment [34].

Cells classically described in the stromal compartment of the intestinal wall are fibroblasts, myofibroblasts, mast cells, macrophages, immune cells and ICC, the latter being considered as the pacemaker cells that regulate gastrointestinal rhythmicity [35]. In recent years, the presence of a new interstitial cell type bearing very long cellular extensions, named telocyte (telos, *i.e*. provided with long-distance cell projections), has been reported in a variety of developing and adult cavitary and non-cavitary organs (http://www.telocytes.com) [11–30,36–43]. To date, a number of studies have described telocytes throughout the entire gastrointestinal tract of humans and laboratory mammals [11–15,40,44,45]. Telocytes are characterized by very long moniliform prolongations, the telopodes, which form a three-dimensional network in close relationship with immune cells, smooth muscle cells, vascular and nervous structures [11–13]. Increasing evidence indicates that telocytes are definitely distinct from the classical fibroblasts, as demonstrated by their peculiar ultrastructural features and differential gene expression and proteomic profiles, as well as microRNA signature [11–13,21–24]. Although telocytes do not possess a unique antigenic profile, at present, CD34, a marker shared with vascular endothelial cells, is the first and the best available choice for the immunohistochemical identification of these cells under light microscopy [12]. Indeed, CD34 expression has been firmly reported in telocytes from different organs [12,14,15,28–30]. Moreover, in the human gut, it has been demonstrated that the CD34-positive interstitial cells are ultrastructurally identifiable as telocytes and correspond to the cells formerly identified as platelet-derived growth factor receptor α-positive ‘fibroblast-like’ cells [14,15]. Therefore, in the present study, we used CD34 as marker for telocytes, and we also performed CD34/CD31, CD34/c-kit and CD34/α-SMA double immunostaining to clearly distinguish telocytes from endothelial cells, ICC and myofibroblasts, respectively. Unfortunately, we could not investigate the ultrastructural features of telocytes by transmission electron microscopy, as archival paraffin-embedded specimens are suitable only for light microscopy.

Following our recent study on telocytes in small bowel CD [28], we herein examined resected left colon specimens from UC patients to evaluate possible differences in the presence and distribution of telocytes in comparison with specimens obtained from controls. The analysis of control samples confirmed the literature description [14,15], with telocytes being located in all the layers of the colonic wall and forming networks in close relationship with other cell types. Interestingly, in sections from UC specimens we observed a severe reduction in telocytes that closely paralleled the fibrotic changes of the colonic wall. In fact, fibrosis was restricted to the muscularis mucosae and submucosa in early fibrotic UC cases, while it extended to affect also wide areas of the muscle layers and the myenteric plexus in advanced fibrotic UC cases. Accordingly, a significant reduction in telocytes was found in the muscularis mucosae and submucosa of both early and advanced fibrotic UC colonic wall. Conversely, while a normal distribution of telocytes was observed in the muscularis propria of early fibrotic UC, the network of telocytes was reduced or even completely absent in fibrotic areas of muscle layers and around myenteric ganglia of advanced fibrotic UC cases. Of note, these data are consistent with those previously reported in disease-affected specimens from CD patients, in which telocytes disappeared particularly in areas where severe fibrosis and architectural derangement of the intestinal wall were observed [28]. Moreover, our group has recently demonstrated that a progressive loss of telocytes occurs in the skin and internal organs of patients with systemic sclerosis, which can be considered a prototypic multisystem fibrotic disorder [29,46]. Finally, evidence for the possible involvement of telocytes in the fibrotic process comes also from animal studies. Indeed, telocytes were found to be decreased during experimental myocardial infarction in rats, particularly in fibrotic areas, and the *in situ* transplantation of cardiac telocytes decreased the infarction size and improved myocardial function [38,47]. Thus, all together these findings point to a possible involvement of telocytes in the fibrotic remodelling of multiple organs, not only the gut. Whether the loss of telocytes might even precede the onset of fibrosis rather than being merely a consequence of the fibrotic process is difficult to be elucidated. However, the findings that in early fibrotic UC cases telocytes were already reduced in the oedematous, less fibrotic areas of submucosa suggest that telocyte loss may be a precocious event during fibrotic remodelling of the intestinal wall.

The role of telocytes in the gut and other organs is still not fully understood, but some potential and relevant functions have been proposed. Accordingly, their loss might have important pathophysiological implications in UC, as previously proposed for CD and systemic sclerosis [28,29,46]. Taking into account the three-dimensional network of telopodes and their strategic position and multiple intercellular relationships within the stromal compartment, telocytes might form a scaffold to guide the migration of other cell types and the correct assembly of the extracellular matrix components, thus contributing to define the correct spatial organization of tissues and organs [12,16,43]. Therefore, the disappearance of telocytes could mechanically contribute to the altered three-dimensional organization of the extracellular matrix during fibrosis. Increasing evidence also suggests that telocytes may participate in intercellular signalling, either directly, by intercellular contacts, or indirectly, by shedding microvesicles and exosomes or secreting paracrine signalling molecules [12,13,20,24–26,48,49]. The excessive deposition of extracellular matrix and the progressive reduction in telocytes may alter the spatial relationships of telopodes with neighbouring immune cells, fibroblasts, smooth muscle cells, ICC and nervous structures possibly impairing intercellular signalling and functions. Telocytes establish intercellular contacts with fibroblasts [26,44,46,50], and thus they might even have a role in the regulation of connective tissue homoeostasis by controlling the activation state of fibroblasts. Thus, it is tempting to speculate that the loss of telocytes might even contribute to promote the transdifferentiation of fibroblasts into profibrotic myofibroblasts. In support to this hypothesis, in the colonic submucosa of our UC patients the disappearance of telocytes was paralleled by the increase in the number of α-SMA-positive myofibroblasts. However, as proposed by some authors [30], we cannot completely rule out the possibility that during pathological processes some telocytes (also referred to as CD34+ stromal cells/fibroblasts/fibrocytes/telocytes) might even change their immunophenotype (*e.g*. loss of CD34 expression and gain of other markers, such as α-SMA), thus contributing to the increase in the myofibroblast population. Nevertheless, our CD34/α-SMA double immunostaining did not reveal the presence of double-positive stromal cells in sections from UC patients, and there has been ultrastructural evidence of telocyte degeneration, rather than activation/transdifferentiation into myofibroblasts, in the setting of fibrosis [46]. Several molecular signalling pathways have been implicated in the differentiation/activation of myofibroblasts [31]. Thus, it will be of major importance to investigate whether the same pathways might be involved in the reduction in telocytes.

In the gut, the three-dimensional network of telocytes has also been proposed to play a specific mechanical and supporting role throughout the different bowel wall layers, being resistant to and deformable following intestine movements [14,15]. Even more importantly, in the muscularis propria telocytes and ICC may form interconnected networks surrounding smooth muscle bundles and myenteric plexus ganglia [12,15,28]. The motility of the gastrointestinal tract involves complex processes that require the structural integrity and functionality of each cellular element involved. Enteric neurons and glial cells, together with ICC, represent the main regulators of motor functions in the gut wall, ensuring coordinated patterns of smooth muscle cell activity [7,51]. It has been suggested that telocytes might be part of this neural/myogenic network and participate in the regulation of gastrointestinal motility, likely by spreading the slow waves generated by the pacemaker ICC [12,15]. This complex neural/myogenic network appears to be markedly altered in UC patients, as shown by severe damages of the enteric neural structures and a reduction in the ICC population, resulting in colonic dysmotility [9,10,52]. In this context, it is noteworthy that in advanced fibrotic UC cases telocytes were significantly reduced in muscle layers as well as in the myenteric plexus, where the network of telocytes was discontinuous or even completely absent around ganglia and in the interganglionic region. In addition, CD34/c-kit double immunostaining clearly revealed that in advanced fibrotic UC telocyte disappearance was paralleled by the loss of the ICC network. On the basis of these findings, we can hypothesize that the reduction in both telocyte and ICC networks in the muscularis propria and myenteric plexus might substantially contribute to colonic dysmotility in UC patients. In support to this hypothesis, both telocytes and ICC have been shown to be decreased in other disorders characterized by intestinal dysmotility, such as CD and intestinal pseudo-obstruction [28,53].

In conclusion, the present findings provide a thorough morphological analysis showing a close relationship between the reduction in telocytes and fibrotic remodelling in the colonic wall of UC patients with long-lasting chronic disease. Because these stromal cells are believed to play multiple roles in the regulation of tissue homoeostasis and functions, including intestinal motility, our data add a new piece to the jigsaw of the mechanisms underlying gut dysfunction in UC. Nevertheless, future functional studies will help to better clarify the role of telocyte loss in the setting of inflammatory bowel disease-associated intestinal fibrosis. Together with previous reports [28,29,38,46,47], the data reported in this study strengthen the idea of a broad involvement of telocytes in tissue fibrosis. Further, the involvement of telocytes (also referred to as CD34+ stromal cells) in pathological processes (*e.g*. tissue repair, fibrosis, cancerogenesis) is increasingly gaining considerable interest [30]. As an example, a reduction in CD34+ stromal cells has been reported in colorectal tumour stroma and peritumoral inflammatory tissue [54]. According to this view, the pivotal goal of a research agenda today should be to dissect the pathogenetic mechanisms targeting telocytes and their roles in different pathologies.
